# Abstracts from German Journals

**Published:** 1872-01

**Authors:** Charles Raushenberg

**Affiliations:** Atlanta, Georgia


### ABSTRACTS FROM GERMAN JOURNALS.

BY CH. RAUSHENBERG, M.D., ATLANTA, GEORGIA.

### *Pathology of the Movable or Wandering Kidney. By Dr. Rudolph H. Ferber, of Hamburg, Germany.*

In this article we find the history of two cases of this comparatively rare pathological occurrence, the principal features of which we shall endeavor to furnish to our readers.

Bernhard Teege, sixteen years old, shoemaker, healthy and well-developed for his age, the son of healthy parents, who worked hard in the stooping posture of his trade up to the time of his sickness, was taken, on September 4th, 1868, with severe pain in the lower abdomen, and burning sensation in the urethra during micturition. Temporary improvement after eight days, but increasing prostration, emaciation, and severe pain in the right inguinal region, became prominent symptoms. He can only sit stooping forward, with the trunk supported posteriorly; can only walk very slowly, with his legs spread out laterally. In the horizontal position, the pain is less severe; but he can only lay on the right side, and then he suffers severely enough to lose much sleep. Inclination to urinate about every two hours, day and night; the act, however, can only be accomplished in a crouching position, and it and that of defecation cause the sensation of hot drops passing along the urethra. Loud talking, sneezing, coughing, etc., occasion severe pain in the right hypochondrium. Pains under both arms and numbness of the left leg are constant.

At the end of November, extreme emaciation, dirty pallor of the skin, morosity, urine of high specific gravity, with profuse sediments of urates, general hyperæsthesia, and abnormal sensations in the urethra, form the characteristic features of the case. No diagnosis.

November 29.—A smooth, uniform tumor, of elastic resistance, is discovered in the left inguinal region, immediately beneath the abdominal integuments in front of the intestines; the hilus is turned upward, the convex edge rests on the ligamentum Poupartii,* and the diagnosis of wandering kidney is established by the plainly visible form of the tumor, and confirmed by the recollection of the patient, who, while engaged in gymnastic exercises in May last, had a severe fall on the back, with fainting, urgent inclinations to defecation, without result during the effort, occasional pain in the region of the kidneys when rising to his feet, and slight inconvenience from deep inspirations, yawning, etc. The excessive tenderness of the tumor prevented any efforts at removing it from its position. Constant gnawing pain at the orifice of the urethra, burning along its course. Urine clear, acid. Micturition easier. A moderate pressure with the flat hand on the tumor, from below upward, relieves the painful tension, particularly when the patient is on his feet; the slightest pressure downward, from obvious reasons, produces excessive pain.

December 4.—Less comfortable, weak, very pale, quite restless. Urine milky, with numerous blood corpuscles. Considerable pain during defecation, relieved by upward pressure on the tumor. Difficult passage of fæces and gases, with the sensation as if a mechanical obstacle seemed to prevent the escape of the latter, and caused them to slip backward again into the intestinal tube. Cutis anserina before defecation. Cutting pains in the rectum. Plumbi acetas 0.06 (four-fifths of a grain)—first thrice and afterwards twice daily—and warm sea-salt baths were used against the pyelitis.

December 7.—Two or three small lumps of pus and pus corpuscles, glued together, followed the evacuation of the

*The same location has as yet been reported only in two cases—those of Braun u Rayer. (Rollet Pathol. and Therap. der Bewegl. Niere Erlangen. 1866. Page 25.)

urine. Urine full of pus. Boring pains under the short ribs of the right side.

December 10.—Sleeps better; urinates every two to three hours. Sensation of formication over different parts of the surface.

December 15.—Kidney lower in the pelvis; hilus nearer the integuments. Pressure upon it causes severe pain at the orifice of the urethra. Inclination to urinate very urgent in the erect position. Urine contains less pus, some blood corpuscles; general hyperæsthesia diminished. Legs less painful; position still stooping. (Drinks fresh beef blood as a nutriment.)

December 21.—More comfortable. Passes, every two hours, about one spoonfull of urine, with blood corpuscles, spirally glued together, so that they are visible to the naked eye; very little pus.

January 10, 1869.—Has used 4.8 (American Pharmacopœia, ℥j.) plumbi acetas; its use is now abandoned. Patient can sit up erect; walks well, and maintains himself in a much better posture. Nutrition and color of skin materially improved; cheerful. Only one edge of the kidney can be felt near the spina ossis ilei superior. Urine contains still some pus, which is generally covered by a profuse rose-colored sediment. Painless defecation twice daily.

January 20.—Attacks of vertigo. Pain, when walking and standing, along the toes, particularly the fifth.

January 24.—Suffers from vertigo every time when rising to his feet; œdem of the face; is able to walk about; has pain across both feet, in a line along the root of the toes, when standing or walking; appetite good; able to work; sometimes slight pains in the upper abdomen; no formication or other nervous symptoms; slight gnawing pain at the orifice of the urethra; urine contains still a little pus; kidney has entirely disappeared; loses his hair. (Wears a tight, elastic abdominal bandage in the shape of bathing drawers, with apertures for the genitals and anus.)

February 17.—Works without pain; perspires freely; has stitches in the left side when walking fast; hardly any pain in the urethra; sleeps sound all night; passes six hundred to eight hundred centimeters* of urine, with sixty centimeters of pus sediment; urinates very seldom—not more than twice or three times daily.

June 15.—Health perfect; nutrition and color of skin faultless; works every day; can walk and dance without any inconvenience; percussion gives a duller sound in the left renal region than in the right; the kidney cannot be discovered through the integuments, but an examination per anus shows the edge of the kidney within reach of the finger on the left side of the rectum. Pressure on that point occasions a sensation as if pressure was exercised externally on the testicle of that side.

Dr. Ferber holds that mechanical disturbances—falls, blows, heavy pressure of the diaphragm and abdominal muscles during labor, etc.—or any concussion or pressure of the renal region, by which the cellular tissue round the kidney and the ligamentnm duodenum-renale are loosened or ruptured, are the common cause of this displacement of the kidney. After once loosened in this manner, the arteria and vena-renalis form the only band by which that organ is connected with any part of the abdomen, and the point where these vessels enter the aorta and vena cava inferior must be the center around which the detached kidney can move as far as the room in the abdominal cavity, the length of the arteria and vena renalis, and that of the urethra, permit such a movement. The kidney, in that condition, according to the laws of gravitation, seeks to gain the lowest point of the abdominal cavity—the small pelvis—where it remains quietly and securely, as it could not be expected ever to ascend upward again to its former normal position.

Cases like this deserve more properly the name of *wandering kidney*. They occur in young subjects, where the walls of the renal vein and artery, peritoneum, etc., are sufficiently elastic and pliable to permit such a distant journey; in older subjects, where these parts offer a greater resistance, the dedetached kikney generally remains higher up in the abdominal cavity, suspended like a pendulum by the renal artery

*One cubic centimeter—equal to sixteen grains of water.

and vein. Rollet's cases, reported from Oppolzer's Clinic, belong to this latter class, which, perhaps more correctly, might be designated as cases of *movable kidney*. Dr. Feiber reports a case which indicates a condition of that kind, as follows:

Miss Charlotte W———, forty-six years old, the child of healthy parents, who died in old age; father and brother of patient are said to have been affected with a disease of the right kidney. Patient has scoliosis lateralis dextra; state of nutrition moderate, color of skin pale yellow. Has menstruated regularly since her seventeenth year. Dates her affliction from a walk performed ten years ago, in anxious excitement, running half a mile. Has suffered, as she states, twice with abdominal inflammation, accompanied with severe pain and vomiting, and has been an invalid during the intervals. Complains of pain in the right hypochondrium, radiating into the genitals and right leg. Experiences some inconvenience in walking and sitting; can only sleep lying on the right side, and has a constant sensation of fulness and tension within the abdomen. Menses profuse, and generally aggravating her trouble. Remains, most of her time, in the horizontal position, and continues to lose flesh. Appetite good, bowels tardy; frequent inclination to urinate. Mental emoions increase the pains. Both kidneys plainly palpable—the left one in situ, the right one much larger, occasionally in the right fossa iliaca, occasionally higher up, nearer to the navel. It is very sensitive to the touch; all efforts at dislocation remain fruitless; it always increases in size during the period of menstruation. Urine frequently contains abundant sediments of urates. Uterus freely movable, not indicating any residues of former pelvi-peritonitis. Menses begin to fail occasionally, and patient always feels materially better during that time, and improves perceptibly as the menses disappear.

As fear and anxiety cause increased secretion of urine, it is probable that a congested state of the kidney, during an unusual bodily exertion, removed one kidney, in this case, from its natural position. The curvature of the spine toward the right side, and the probable predisposition to disease of the right kidney, account for the occurrence of the affection on that side. The intestinal inflammations spoken of above were probably not inflammations, but symptoms of strangulation, occasioned by the enlarged kidney in its progress downward.

Amongst fifty-nine cases of this disease, reported in different places, only nine occurred in males. The much more frequent occurence of it in females who have undergone several deliveries seems to indicate respectively a loosening and a rupture of the ligaments, keeping the kidneys in situ during the process of labor.

The statement of Rosenstein—"The principal occurrence, which first attracts the attention and excites the fear of the patient in this disease, is a tumor in the abdomen, which suddenly, after some accidental cause, protrudes beyond the free edge of the ribs"—is not applicable to all cases, as those reported above prove.

A considerable derangement of nutrition, emaciation and loss of strength constituted the principal phenomena of our first case. They were not the consequences of profuse suppuration, as pyelitis did not make its appearance until the 4th of December, three months after the commencement of grave symptoms, but of intense pain, disturbed sleep and general nervous irritation in a young man in the period of development.

A correct diagnosis is only possible after the appearance and recognition of the kidney as such out of situ at different abnormal localities in the abdomen. A concussion of the renal region, followed by pyelitis, does not justify the diagnosis of movable or wandering kidney.

I have seen a case in a lady twenty-six years old, where only a catarrh of the renal calices could be demonstrated, suffering with manifold renal troubles, which the patient all referred to a severe horseback ride and a fall from a sofa. After the existence of pyelitis for several years, hydronephrosis proved the kidney, which before could not be felt through the integuments, in situ.

Dr. Ferber recommends plumbum aceticum for the pyelitis, as a reliable astringent, which does diminish the formation of pus perceptibly. In the second case reported above, he gave

2.52 (thirty-seven grains) plumb. acet. within four weeks. The quantity of pus diminished very decidedly, the reflex phenomena on the part of the stomach and the intestines improved at least temporarily, and the diarrhœal evacuations were checked.

The use of beef's blood, fresh from a slaughtered animal, as a nutriment, has been used with decided advantage in these and other cases; also warm sea-salt baths. The beef's blood can, of course, only be used with patients who live near a slaughter-house, where animals are killed several times every week. It is generally taken warm, and most patients soon overcome the repugnance which they experience when they begin to use the remedy. Drinking it in a dark room, or out of a non-transparent vessel, alleviates this difficulty. A drink of claret is generally taken after the blood. This remedy has also been used with evident advantage in rhachitic children.—*Virchow's Archiv., January*, 1871.

---

*Contributions to the Knowledge of Pharyngeal Diphtheritis. By Dr. A. Classen, at Rostock, Germany.*

This able and interesting article contains the results of the writer's experience, derived from the observation and treatment of one hundred and fifty-five cases of this disease, during a period of time extending from October, 1862, to March, 1870, at Rostock, the capital of Mecklenburg Schwerin, in the north of Germany.

Of the thirty-seven deaths which occurred in the one hundred and fifty-five cases, twenty-seven perished by suffocation from transmission of the disease to the respiratory organs; two from affections of the small bronchia after tracheotomy; eight by inanition, or infection of the blood; one hundred and eighteen recovered. Of all the patients thirty-one were past twenty years old; twenty-six were between ten and twenty years; ninety-eight were under ten years. In four cases, where tracheotomy was considered necessary, but not performed on account of the unwillingness of the parents, recovery took place without it.

Among the twenty-seven cases mentioned above, which died from transmission of the disease to the larynx, were nineteen children under two years old. They all died between the second and fourth day after having been taken. Not one child of that age, affected in this manner, got well. Two cases of simple croup in children of that age, who recovered under the use of emetics and mercurials, I did not include in the above numerical statement, as not the slightest sign of diphtheritic exudations could be discovered in the pharynx. The other eight cases among the twenty-seven mentioned above, occurred in children between the fourth and tenth year. The disease lasted from six to fourteen days. The fever was only high in the first two days; afterwards mild, but continuous, prostration, obstinate epistaxis, apathy and death. Of the eight cases of death by inanition, etc., six were children, the youngest three, the oldest fourteen years old. In four cases the course of the disease corresponded with the above described image, where, with a disfigured countenance, a swelled neck, a cadaverous fetor, the deepest apathy, which rejects every aid and all nourishment, prevailed early in the disease. The pulse was moderately frequent, irregular, and death occurred with loose tracheal rattles, but not by firm stenosis of the larynx.

Other cases, representing death by inanition or blood-infection still more strikingly, are the following: An eleven-year-old girl was apparently perfectly convalescent. Large flakes of false membrane were detaching themselves from the tonsills and velum palati; appetite improving. All at once, while raising up in bed, she fell back dead. Another was a stout laborer, twenty years old. Exudation on the tonsils very moderate; much pain in the throat; fetor very disagreeable; enormous enlargement of the glands of the neck; increase of the already considerable fever on the fourth day; very small and irregular pulse; sopor; consciousness clear, when aroused; disposition cheerful when talking, generally remaining quiet and apathetic; died suddenly, with moderate mucous râles, after having made several deep inspirations, connected with vesicular respiration, a very short time before his death.

To give a normal image of the usual course of the disease is impossible, as the different cases present too great a variety of forms. The lightest cases occurred in the relatives and room-mates of the graver patients. These cases would frequently have been overlooked, or remained unnoticed, under different circumstances, as they consisted, generally, in a few small white patches on the tonsils and velum palati, with little or no fever and light catarrhal symptoms. Their duration was confined to two or four days.

Another class of cases are those which remain unnoticed for a few days, until, all at once, with the occurrence of graver symptoms, large diphtheritic spots are discovered in the pharynx. The purely local character of the primary affection accounts for this occurrence. Fever and swelling of the lymphatic glands of the neck frequently, are the first symptoms which reveal the true state of things.

A third group of cases characterizes itself by the high degree of fever connected, from the commencement, with the pharyngeal troubles, and by a return to health as sudden as their beginning. They seldom occupy more than three or four days. In a grown man diphtheritic patches made their appearance on the tonsils, and almost at the same time a large bulla, filled with a turbid fluid, appeared on one leg above the tibia, which, after bursting, exhibited a diphtheritic coat on the corium, notwithstanding the patient was free of fever and pseudo membranes, in four days. The swelling of the lymphatic glands of the neck is not absent in these cases.

Among the one hundred and eighteen recoveries, thirty-eight belonged to the second, and fifteen to the third group mentioned above, and the other sixty-four cases lasted from six to fourteen days, and were followed by paralysis, or other consecutive diseases.

The microscopic examination of false membranes by Dr. Classen, leads him to the final conclusion that they contain no new formation—irrespective of the immigrated fungi, or fungus-like bodies—except epithelial cells, the layers of which are separated by other layers consisting of nuclei, blood and pus corpuscles, in a coagulated, fibrinous substance.

The fine, glittering globules which he observed so abundantly, principally in the upper epithelial layer of false membranes, and which, magnified seven hundred and fifty times, did not assume a definite shape, he does not consider a product of the organism, but identical with the so-called swarmers of Hüter, Schônborn and Tomassi. They have frequently been observed in lively upward and downward motion, and were first seen in the blood of patients with hospital gangrene, and afterward in those afflicted with diphtheritis. They were exposed by Dr. Classen to the influence of acetic acid, iodine, tincture of pepsin, liquor potassæ, caustic and ether, but remained entirely unchanged, showing that they can not be protein, or fat molecules, but must be cellulose formations, fungus-like organisms. They settle first in the exterior epithelial layer of the mucous membrane, and migrate from there in consequence of their immense smallness and, probably, their independent motion in the deeper parts, and through the circulation into the tissues generally. Classen looks upon these minute foreign bodies as the originators of the disease, their first appearance and multiplication in the epithelial cells of the mucous membrane as the primary local lesion and the hearth of infection of the whole system, and the cause of the consecutive secondary symptoms. While he admits the occasional occurrence of deep ulceration of the mucous membrane beneath the exudations, he does not consider this the rule, but says that the ulcers are generally shallow, and do not require suppuration, but heal similarly to ulcers of the cornea, by restitution of the normal epithelium.

Croup, he thinks, is, if not anatomically, at least clinically, different from diphtheria, because in croup the false membrane is the simple product of a high-graded catarrhal inflammation of a mucous membrane, not often connected with necrotic decay, and not followed by any other blood-changes but those produced by simple inflammatory products, while in diphtheria the false membrane is the product of specific local irritation of the mucous membrane by those minute fungi-form foreign bodies, which, in consequence of their introduction in the blood, is frequently followed by a peculiar blood-fermentation characterized by symptoms indicating a grave general lesion.

The infection of the blood in diphtheria corresponds by no means with the degree of the fever. It frequently lasts much longer than the fever and the local inflammation. The former seems principally caused by the inflammation of the mucous membrane and the lymphatic glands. Those cases which presented most strikingly the image of blood-poisoning did not show a high degree of fever, but a decidedly cool skin. Disposition to hemorrhages and specific inflammations in the respiratory organs, particularly the capillary bronchi, seem, according to several striking observations of our writer, to be one of the most frequent consequences of the altered condition of the blood, and one of the most frequent causes of death.

The occurrence of paralysis in this disease, Classen refers, with Buhl,* to hemorrhagic effusion into the neurilema, and reports a case, in illustration of this view, where a fifteen-year-old boy, who had recovered from a severe and tedious laryngeal diphtheria, after suffering a while from deafness, experienced a considerable diminution of the faculty of sight, (imperfect vision,) with glimmering before the eyes and piercing pains in the depth of the eye-balls. He considers this defect the consequence of an effusion into the sheath of the optic nerve near its origin—the only case of that kind reported as a sequence of diphtheria.

The morbid condition of the digestive organs in diphtheria he represents as effects of the necrotic substances, which enter the stomach when particles of false membranes are swallowed, and uses diluted aqua chlor. for the correction of these symptoms.

He refers to the relation existing between scarlatina and diphtheria, and to the fact which has several times come under my own observation in this country—that while one child had scarlatina, several others of the same family had diphtheria soon before or afterwards.

An early destruction of the local fungoid infection of the mucous membrane by therapeutical measures, he considers the first rational indication. While this intention has been manifested in the treatment of this disease by many physicians, its realization has not yet been attained, as the forms

* Zeitschrift für Biologie, Vol. III, p. 351.

of the applications for that purpose are often incompatible with other conditions of the organism. Concentrated mineral acids, in consequence of their destructive effect on the mucous membrane, are inadmissible. Chromic acid, as recommended by Lewin, is the most available agent of all, as it is tolerably well borne in sufficient dilution, but it often happens that the irritablity of the mucous membrane forbids its further use before all the deleterious substances are destroyed. Cauterizations with argentum nitricum, which have been extensively used by German physicians, have generally proved unavailable in all but the lightest forms of the disease. Liquor ferri sesqui chlorati, early and repeatedly used on small diphtheritic membranes, frequently gives a favorable turn to the condition of affairs; but as this change occurs as often without any local treatment, and as it has, in spite of its most energetic application, often failed in very light cases to hasten the detachment of the false membranes, it still must be considered a remedy of doubtful efficacy.

Among all remedies of that class the milder acids, according to theory and experience, seem to merit a decided preference. Inhalations of lactic acid, where the affection of the larynx and trachea are not too extensive, has proved of considerable power in the destruction of the local morbid poison. Citric acid has, for the same reasons, been used, and seems to exercise a very beneficial influence to the same effect, particularly if it is used according to the method so warmly recommended by Révillout and Trousseau. It is not sufficient to use the lemon juice externally every hour, but the patient must eat the pulp of about four lemons every hour for several days in succession. Dr. Révillout reports that he has successfully treated his own case by this remedy after having been abandoned as hopeless, and that he has used it ever since with great success. The use of this remedy in children is very difficult.

Amongst all acids which have a tendency to destroy fungous organizations, the sulphurous acid seems to merit the preference, not only on account of its undoubted chemical effect, but also its easy and simple form of application. Dr. Alban Lutz, of Munich, has the merit of having introduced this important remedy. He uses the flores sulphuris by blowing them into the pharynx and larynx, and gives it also internally, made into a mixture. There exists no doubt that sulphurous acid is continually developed from the dry flowers of sulphur, and much more when they are in permanent contact with a moist mucous membrane, and also no doubt that this acid has a destructive effect on the various organisms of a lower order. Its momentary effect upon the mucous membrane is very mild; but it continues, and, therefore, can be maintained by keeping it on the surface of these membranes any desired length of time. Dr. Classen did not lose one patient out of fourteen within four months after he commenced using this treatment—quite an unusual success in comparison with his former experience; but two fatal cases, immediately afterward, convinced him that his remedy was not infallible. It cannot be expected that a sufficient amount of sulphurous acid enters the blood to prevent death by secondary lesions. Upon the whole, however, sulphurous acid is considered a decided progress in the local treatment of diphtheria.

The other antiseptic remedies—liquor sodæ carbol., potass permanganate, aqua chlor., etc.—are either not efficient within themselves, or cannot be used long enough. In light cases, they seem to be useful occasionally—particularly as gargles for prophylactic purposes—but they can never prevent an unfavorable issue in severe cases. Aqua calcis, as an antiseptic and anti-catarrhal remedy in inhalations, where it becomes necessary to reach the larynx, is a useful remedy in mild cases. Alum, potass chlor., sulphas zinci, in weak solutions, can be made useful against the hyperæmia existing in diphtheria, but are powerless in arresting the progress of the exudation. Local abstractions of blood, where the local inflammations are severe, are neither theoretically nor practically objectionable. Mercurials, particularly in the form of external frictions of unguentum mercuriale to the neck, are no real antidote, but have often been used with a happy effect; and A. von Gräefe's classic essay on the treatment of diphtheritic conjunctivitis, has clearly demonstrated the rationality and usefulness of mercurials in this disease, and caused

Dr. Classen to use them, and with remarkably favorable results in a majority of cases.

As we do not yet know of a remedy by which the blood-changes occurring in this disease are normalized, diphtheria does not become curable. Quinine is a useful sedative and antipyretic, lessening the fever and sustaining the nerve-power, and it as well as iron deserves high commendation in the treatment of the disease; but it is very doubtful whether they have a normalizing effect upon the specific alteration of the blood in these cases, while they are very certainly of great importance in the period of convalescence.

Governed by the idea that the fungoid bodies are the originators of the abnormal condition of the blood in diphtheria, the writer of this article has lately given chrystalized carbolic acid, in the form of powder, with very marked improvement of the symptoms. These observations, however, are not yet numerous enough to be considered of great weight, but this remedy deserves a thorough trial.—*Virchow's Archiv.*, *February*, 1871.

